# Structural equation model analysis of promoting physical activity behavior among college students: based on control-value theory of achievement emotions

**DOI:** 10.3389/fpsyg.2025.1669445

**Published:** 2025-11-20

**Authors:** Han Li, Tao Fang, Xinbo Wu, Zhenqian Zhou, Xilong Wu, Zhengxiao Zhang, Sukwon Kim

**Affiliations:** 1 College of Physical Education, Anhui Normal University, Wuhu, Anhui, China; 2 College of Sport, Exercise and Health Sciences, Loughborough University, Loughborough, United Kingdom; 3 School of Artistic Sport, Hunan Agricultural University, Changsha, China; 4 Department of Physical Education, Jeonbuk National University, Jeonju, Republic of Korea

**Keywords:** control-value theory of achievement emotions, physical activity, behavior, structural equation model, college students

## Abstract

**Background:**

Prior studies have examined various determinants of college students’ physical activity behaviors; however, limited attention has been given to the role of a supportive environment within physical education courses. According to Pekrun’s control-value theory of achievement emotions, individuals’ appraisals of control and value in specific learning contexts evoke corresponding achievement emotions, which subsequently influence their learning behaviors and outcomes.

**Objective:**

This study aimed to examine how a supportive teaching environment in physical education classes influences college students’ physical activity behaviors through control-value appraisals and achievement emotions, contributing to a broader application of the control-value theory of achievement emotions.

**Method:**

A total of 1,562 college students from five universities in China completed questionnaires, including the Physical Education Demand Support Questionnaire, Academic Control Scale, Subjective Task Value Scale of Sports, Achievement Emotions Questionnaire, and Physical Activity Rank Scale-3. Structural equation modeling (SEM) was used to validate the relationships.

**Results:**

Control-value appraisals and two achievement emotions—enjoyment and boredom—played partial mediating roles in the relationship between a supportive teaching environment and college students’ physical activity behaviors. The chain indirect effects through control appraisal and enjoyment, and through control appraisal and boredom, were 0.022 and 0.008, respectively. Similarly, the indirect effects through value appraisal and enjoyment, and through value appraisal and boredom, were 0.034 and 0.010, respectively.

**Conclusion:**

Teacher autonomy support indirectly influenced college students’ physical activity behaviors through control-value appraisals and achievement emotions, particularly enjoyment and boredom. Enjoyment emerged as an important achievement emotion that positively shaped exercise motivation and behavior, highlighting the critical role of emotional experiences in linking supportive teaching environments with students’ physical activity. These findings extend the control-value theory of achievement emotions to the context of physical education and provide empirical evidence for promoting teacher autonomy support to foster students’ exercise engagement.

## 1 Introduction

Regular participation in physical activity offers a wide range of benefits for college students, including improved physical fitness, enhanced mental health, better academic performance, and increased social interaction and cohesion (Rehman et al., 2024; Teferi, 2020; Gilchrist and Wheaton, 2017). Despite these advantages, recent social transformations and rapid technological advancements have significantly altered students’ lifestyles, leading to changes in their health behaviors (Peterson et al., 2018). Many college students now face increasing academic pressures and spend extended hours on digital media, contributing to sedentary behaviors and insufficient engagement in physical activity (Deliens et al., 2015).

Reflecting on prior research, scholars have analyzed the determinants of college students’ physical activity behavior, considering both internal psychological factors and external environmental factors, such as exercise self-efficacy, exercise attitudes, availability of campus facilities, and familial support (Cheng, 2024; Zhang et al., 2022). However, physical education classes, as a vital context for shaping students’ exercise attitudes and emotional experiences, have received relatively little attention. The autonomy-supportive teaching environment established by teachers may represent a crucial contextual factor in promoting students’ sustained engagement in physical activity, yet this area remains underexplored.

Previous studies have predominantly drawn on self-determination theory and expectancy-value theory to examine the influence of physical education classes on students’ motivation and physical activity behavior (Martin and Kulinna, 2004; Fletcher, 2016). However, these theories emphasize the motivation but pay relatively little attention to students’ emotional experiences during PE and how these emotions affect subsequent exercise behaviors. Therefore, it is necessary to introduce a theoretical framework that systematically explains the generation of emotions and their relationship with behavior.

The control-value theory of achievement emotions (CVTAE) provides a comprehensive framework for understanding how emotions influence learning and behavior (Pekrun, 2006). Central to this theory is the control-value appraisal, which determines the type and intensity of achievement emotions and reflects students’ motivational beliefs (Pekrun, 2006). Within this framework, control beliefs refer to individuals’ perceived ability to influence performance and outcomes, whereas value beliefs concern the perceived importance or worth of the task. Value beliefs are typically divided into intrinsic value, which relates to the inherent enjoyment and meaning of the activity, and extrinsic value, which pertains to its instrumental or practical benefits (Frenzel et al., 2023).

Building on these foundations, Pekrun and colleagues proposed that autonomy support—a key aspect of the social learning environment—plays a significant role in shaping students’ control and value appraisals (Pekrun, 2006). When teachers foster autonomy by encouraging choice, independence, and personal responsibility, students are more likely to experience higher self-efficacy and perceive greater value in learning tasks (Pekrun, 2024). In PE settings, autonomy-supportive teaching has been shown to enhance students’ positive emotions and promote long-term participation in physical activity (Chan and Lam, 2023). However, relatively little is known about how teacher autonomy support interacts with students’ control-value appraisals to generate specific achievement emotions in PE contexts.

According to Pekrun’s classification, achievement emotions can be distinguished as activity-related or outcome-related (Camacho-Morles et al., 2021). Activity-related emotions—such as enjoyment, boredom, and anger—arise during the engagement in an activity and reflect students’ immediate affective experiences. Outcome-related emotions, including hope, anxiety, and shame, are typically associated with anticipated or actual results. Because physical education emphasizes active participation, interpersonal interaction, and experiential learning, activity-related emotions are particularly relevant for understanding students’ in-class experiences and their subsequent exercise behaviors. Consequently, the present study applies the CVTAE to investigate how teachers’ autonomy support influences students’ activity-related emotions and behavioral engagement in PE through control-value appraisals, thereby uncovering the emotional mediation mechanisms underlying students’ physical activity behaviors.

Although previous studies have explored the application of the CVTAE in educational and sport contexts (Li, 2021), several important gaps remain. First, few have simultaneously examined the interplay between teacher autonomy support at the contextual level and control-value appraisals at the individual level within PE settings. Second, much of the existing work has focused on general affective states rather than discrete emotions that may differentially predict sustained engagement in physical activity (Simonton, 2021). To address these limitations, this study develops an integrative model grounded in the CVTAE, illustrating how autonomy-supportive teaching environments influence college students’ exercise behaviors through control-value appraisals and achievement emotions processes. Furthermore, both control-value appraisals and achievement emotions are shaped by cultural factors (Pekrun, 2024). In the Chinese collectivist context, students’ perceptions of control and value are deeply intertwined with social expectations, interpersonal harmony, and collective goals (Chan and Lam, 2023). These socio-cultural values may highly connected with teacher support and peer relationships on emotional experiences in PE. By focusing on Chinese college students, this study also extends its cross-cultural applicability to an East Asian educational context.

Given that the CVTAE involves multiple mediating processes and latent constructs, structural equation modeling (SEM) was employed as an appropriate analytical approach. This method allows for simultaneously examination of the complex relationships among environmental, cognitive, emotional, and behavioral variables (Hair et al., 2021). This approach enables a comprehensive test of the proposed model, offering both theoretical insights and empirical evidence for promoting autonomy-supportive teaching as a pathway to enhance students’ sustained participation in physical activity.

## 2 Hypotheses

This study develops a hypothetical model of physical activity-promoting behavior among college students, anchored in the CVTAE. Within this model, academic control belief and subjective task value belief are employed to represent control and value appraisals, respectively. Academic control belief reflects control evaluation through the student’s perceived ability and confidence to complete a learning task (Simonton et al., 2021). Meanwhile, subjective task value belief represents value evaluation, encompassing the perceived usefulness and appeal of the learning task (Shen et al., 2024). Drawing on previous research concerning the physical education classroom environment, teacher’s autonomy support is included as a critical element representing the learning environment (Zimmermann et al., 2021; Wang et al., 2024). Therefore, this study adopts a similar approach. Physical activity behavior is used to reflect the long-term effects of achievement in youth sports, analogous to academic achievement in traditional educational settings (Li, 2021). Enjoyment, boredom, and anger are chosen as achievement emotions, representing positive and negative, activation and deactivation-related activity emotions, respectively (Simonton et al., 2021). Building on the aforementioned theoretical framework, we propose the following hypotheses to explore the relationships among various variables:

*H1*: Teacher’s autonomy support is a positive predictor of college students’ academic control belief and subjective task value belief.

*H2*: College students’ academic control belief and subjective task value belief positively predict classroom enjoyment, while they negatively predict classroom boredom and anger.

*H3*: Classroom enjoyment positively predicts physical activity behavior, whereas classroom boredom and anger negatively predicts physical activity behavior.

*H4*: Teacher’s autonomy support is indirectly associated with classroom enjoyment, boredom, and anger through students’ academic control belief and subjective task value belief.

*H5*: College students’ academic control belief and subjective task value belief are indirectly associated with physical activity behavior through classroom enjoyment, boredom, and anger.

*H6*: Teacher’s autonomy support is indirectly associated with physical activity behavior through students’ academic control belief, subjective task value belief, classroom enjoyment, boredom, and anger.

## 3 Methods

### 3.1 Participants

According to the heuristic guidelines proposed by Kline and Hair, the recommended sample size for structural equation modeling (SEM) is approximately 10–20 participants per estimated parameter, with more complex models requiring larger samples. The proposed model in this study includes around 125 free parameters, suggesting that a sample size of approximately 1,250–2,500 participants would be needed to ensure stable parameter estimation. This study was conducted in March 2024 across five undergraduate universities located in Changsha City, Hunan Province, China, and all participants signed informed consent forms approved by the Hunan Normal University Ethics Committee (Approval ID: 2024646). All participants in the study provided informed consent prior to their involvement. Given that physical education classes are typically scheduled during the first and second years of university, two administrative classes from each year were randomly selected at five universities, comprising a total of 1,562 college students. With the teacher’s assistance, the questionnaire survey was administered under the supervision of the researches and collected on-site. Out of 1,562 distributed questionnaires, 1,530 were retrieved, yielding a recovery rate of 97.95%. Following collection, validity checks were performed, and 42 questionnaires were discarded due to incomplete answers or evident errors, resulting in 1488 valid questionnaires and an effective response rate of 95.26%. The demographic breakdown included 769 males (51.7%) and 719 females (48.3%), with 754 first-year students (50.7%) and 734 s-year students (49.3%). The average age is (19.51 ± 1.295) years old. All participants were typically enrolled in physical education classes and reported no physical illnesses.

### 3.2 Measurements

#### 3.2.1 Teacher’s autonomy support

The study employed the Physical Education Demand Support Questionnaire developed by Yin et al. (2018), which has undergone rigorous professional reliability and validity verification. This scale has been widely used among Chinese college students (Lv and Zhang, 2024; Yuan et al., 2025). This scale comprises six items, utilizing a Likert 7-point scoring method that ranges from “completely disagree” (1 point) to “completely agree” (7 points). The scores from each item are aggregated, with higher totals indicating greater autonomy support from university physical education teachers. Confirmatory factor analysis (CFA) supported a one-factor structure, with all six items retained for the final SEM analysis. The model fit indices were satisfactory: *χ*^2^/df = 3.760, RMR = 0.030, NFI = 0.994, GFI = 0.993, CFI = 0.995, and RMSEA = 0.043. The scale demonstrated excellent internal consistency (Cronbach’s *α* = 0.913) and satisfactory composite reliability (CR = 0.91).

#### 3.2.2 Academic control belief

The Academic Control Scale developed by Perry et al. (2001) was utilized for measuring students’ perceptions of control. This scale was translated into Chinese and adapted for the physical education context by Li (2021), and its reliability and validity have been professionally tested and confirmed. This scale has been widely used among Chinese college students (Su et al., 2025; Huang and Zhang, 2025). This scale includes 8 items, employing a Likert 5-point scoring method ranging from “completely disagree” (1 point) to “completely agree” (5 points). Four of the eight items are negatively worded and therefore reverse coded. The total score is calculated by summing the points from each item, with higher scores indicating a stronger sense of academic control among college students in sports settings. CFA supported a one-factor structure. The model demonstrated excellent fit indices: *χ*^2^/df = 4.744, RMR = 0.022, NFI = 0.985, GFI = 0.984, CFI = 0.988, and RMSEA = 0.050. The scale demonstrated excellent internal consistency (Cronbach’s *α* = 0.914) and satisfactory composite reliability (CR = 0.91).

#### 3.2.3 Subjective task value belief

The Subjective Task Value Scale of Sports, developed by Xiang et al. (2003), was utilized for assessment. This scale has been widely used among Chinese students (Zhang and Li, 2023). This scale comprises a total of 6 questions. It employs a Likert 7-point scoring method, ranging from “completely disagree” (1 point) to “completely agree” (7 points). Scores are aggregated across items, with higher totals indicating a greater perceived value of sports among college students. CFA supported a one-factor structure. The model demonstrated excellent fit indices: *χ*^2^/df = 3.639, RMR = 0.023, NFI = 0.995, GFI = 0.993, CFI = 0.997, RMSEA = 0.042. The scale demonstrated excellent internal consistency (Cronbach’s *α* = 0.936) and satisfactory composite reliability (CR = 0.937).

#### 3.2.4 Achievement emotions

Achievement emotions were assessed using the Achievement Emotions Questionnaire (AEQ) developed by Pekrun et al. (2011), which includes three discrete achievement emotions evaluation subscales: classroom enjoyment, classroom boredom, and classroom boredom. The classroom enjoyment subscale comprises four questions, the classroom boredom subscale includes 10 questions, and the classroom anger subscale comprises five questions. This scale was also translated into Chinese and adapted for the physical education context by Li (2021), and its has been widely used among Chinese college students (Chang, 2024). AEQ utilizes a Likert 5-point scoring method, ranging from “completely disagree” (1 point) to “completely agree” (5 points). Scores from each subscale are aggregated, with higher scores indicating stronger classroom achievement emotions among college students. The model displayed robust fit indices: *χ*^2^/df = 3.173, RMR = 0.028, NFI = 0.974, GFI = 0.965, CFI = 0.982, RMSEA = 0.038. Additionally, the Cronbach’s alpha for the subscales were 0.886, 0.936, and 0.918 respectively, the CR was 0.884, 0.936, 0.915, respectively. The scale demonstrated excellent internal consistency and satisfactory composite reliability.

#### 3.2.5 Physical activity behavior

The Physical Activity Rank Scale-3 (PARS-3) was employed to assess respondents’ participation in mass sports over the past month (Duan et al., 2022), which has been widely used among Chinese students (Wu et al., 2024). PARS-3 evaluates three dimensions of physical activity: intensity (“What level of physical exertion do you engage in?” with scores ranging from 1 to 5), duration (“How many minutes at a time do you engage in the described physical activity?” with scores ranging from 1 to 5), and frequency (“How frequently do you participate in the aforementioned physical activities?” with scores ranging from 0 to 4). The overall PARS-3 score is calculated by multiplying the intensity, duration, and frequency scores, resulting in a total score that can range from 0 to 100. The level scale for this study is categorized as low (less than 20 points), medium (20–42 points), and high (greater than 42 points). The Cronbach’s alpha for the study population is 0.775.

### 3.3 Statistics

Prior to data analysis, missing data were examined and treated. Cases with more than 5% missing responses were excluded from the final dataset (*n* = 42). Tests of univariate and multivariate normality were conducted. Skewness and kurtosis values for all variables ranged between −1.5 and +1.5, and Mardia’s coefficient indicated acceptable multivariate normality. These results justified the use of maximum likelihood estimation in SEM.

For the analysis of Structural Equation Modeling (SEM) in this study, we utilized AMOS 26.0 software. To assess the factor structure and internal consistency of the scales used, we conducted Confirmatory Factor Analysis (CFA) and estimated Cronbach’s alpha coefficients. The CFA evaluation metrics revealed a *χ*^2^/df ratio of less than 5, with Comparative Fit Index (CFI), Normative Fit Index (NFI), and Goodness of Fit Index (GFI) values all exceeding 0.90. The Root Mean square Residual (RMR) and Root Mean Square Error of Approximation (RMSEA) were less than 0.08. These results indicate that the model fit was robust and met established adequacy standards (Gholami Fesharaki, 2020).

After establishing validity and reliability, descriptive statistics, correlation estimates, and regression analysis of the primary variables were conducted using IBM SPSS Statistics (version 25.0). We employed Harman’s one-factor test to assess common method bias by subjecting all items to an unrotated principal component factor analysis (Aguirre-Urreta and Hu, 2019). Subsequently, AMOS 26.0 software was used to construct the SEM model. Initially, the model fit indices and the relationships between each path factor were assessed. We then evaluated the indirect effects of the influencing factors using the bias-corrected bootstrap estimation method with a 95% confidence interval. An indirect effect was considered statistically significant if the confidence interval of the path coefficient did not include zero.

## 4 Results

### 4.1 Common method bias

Harman’s one-factor method was employed to assess common method bias. The results indicate that the initial eigenvalues of eight factors exceed 1, with the first factor explaining 26.433% of the variance, which falls below the critical threshold of 40%. To further evaluate this, we performed a single-factor confirmatory factor analysis using AMOS 26.0, where all items were loaded onto a single-factor model. The resulting model fit indices were as follows: *χ*^2^/df = 33.638, RMR = 0.262, NFI = 0.345, GFI = 0.354, CFI = 0.351, and RMSEA = 0.148. These findings indicate that no single factor dominated the variance. This suggests that common method bias was unlikely to have substantially influenced the results, ensuring the credibility of subsequent analyses.

### 4.2 Descriptive statistics and correlations estimates

Table 1 clearly illustrates significant correlations between several variables. Physical activity behavior, teacher’s autonomy support, academic control belief, subjective task value belief, and classroom enjoyment were significantly positively correlated with each other. Conversely, classroom boredom and anger were significantly negatively correlated with other elements. These results indicate that more supportive teaching environments, stronger control–value appraisals, and positive emotions are associated with higher engagement in physical activity. The correlation patterns provide preliminary support for the hypothesized model.

**Table 1:** Descriptive statistics and correlations estimates.

Variables	M	SD	1	2	3	4	5	6	7
1 Support	22.74	8.04	1						
2 Control	27.00	7.21	0.251**	1					
3 Value	22.39	8.21	0.283**	0.332**	1				
4 Enjoyment	13.97	3.80	0.224**	0.255**	0.287**	1			
5 Boredom	34.13	9.12	−0.167**	−0.244**	−0.245**	−0.131**	1		
6 Anger	16.39	5.04	−0.374**	−0.365**	−0.493**	−0.330**	0.230**	1	
7 Exercise	30.14	24.56	0.160**	0.119**	0.166**	0.323**	−0.136**	−0.176**	1

Support = teacher’s autonomy support, control = academic control belief, value = subjective task value belief, exercise = physical exercise behavior, ** indicates *p* < 0.01.

### 4.3 Regression analysis

In this study, gender, age and grade were used as control scalars, regression analysis was used to explore the predictive effects of teacher’s autonomy support, academic control belief, subjective task value belief, classroom enjoyment, boredom, and anger on physical activity behavior of college students. Table 2 reflects the results of regression analysis. The results show that different prefactors can significantly predict the physical activity behavior of college students. It was found that classroom enjoyment was the strongest predictor of college students’ physical activity amount, which could explain 10.2% of physical activity variation.

**Table 2:** Regression analysis.

Variable	Model 1	Model 2	Model 3	Model 4	Model 5	Model 6	Model 7
Gender	−0.010	0.010	0.001	0.009	0.004	−0.005	0.012
Age	0.028	0.027	0.012	−0.004	−0.005	0.013	0.001
Grade	−0.012	−0.023	−0.016	−0.022	−0.023	−0.013	−0.038
Support		0.162***					
Control			0.118***				
Value				0.17***			
Enjoyment					0.324***		
Boredom						−0.134***	
Anger							−0.183***
*R* ^2^	0.001	0.027	0.014	0.028	0.105	0.019	0.032
*F*	0.498	10.179***	5.431***	10.727***	43.328***	7.087***	12.412***
*△R^2^*	0.001	0.024	0.012	0.025	0.102	0.016	0.03

Support = teacher’s autonomy support, control = academic control belief, value = subjective task value belief, exercise = physical exercise behavior, *** indicates *p* < 0.001.

### 4.4 Structural equation model fit

**Figure 1:**
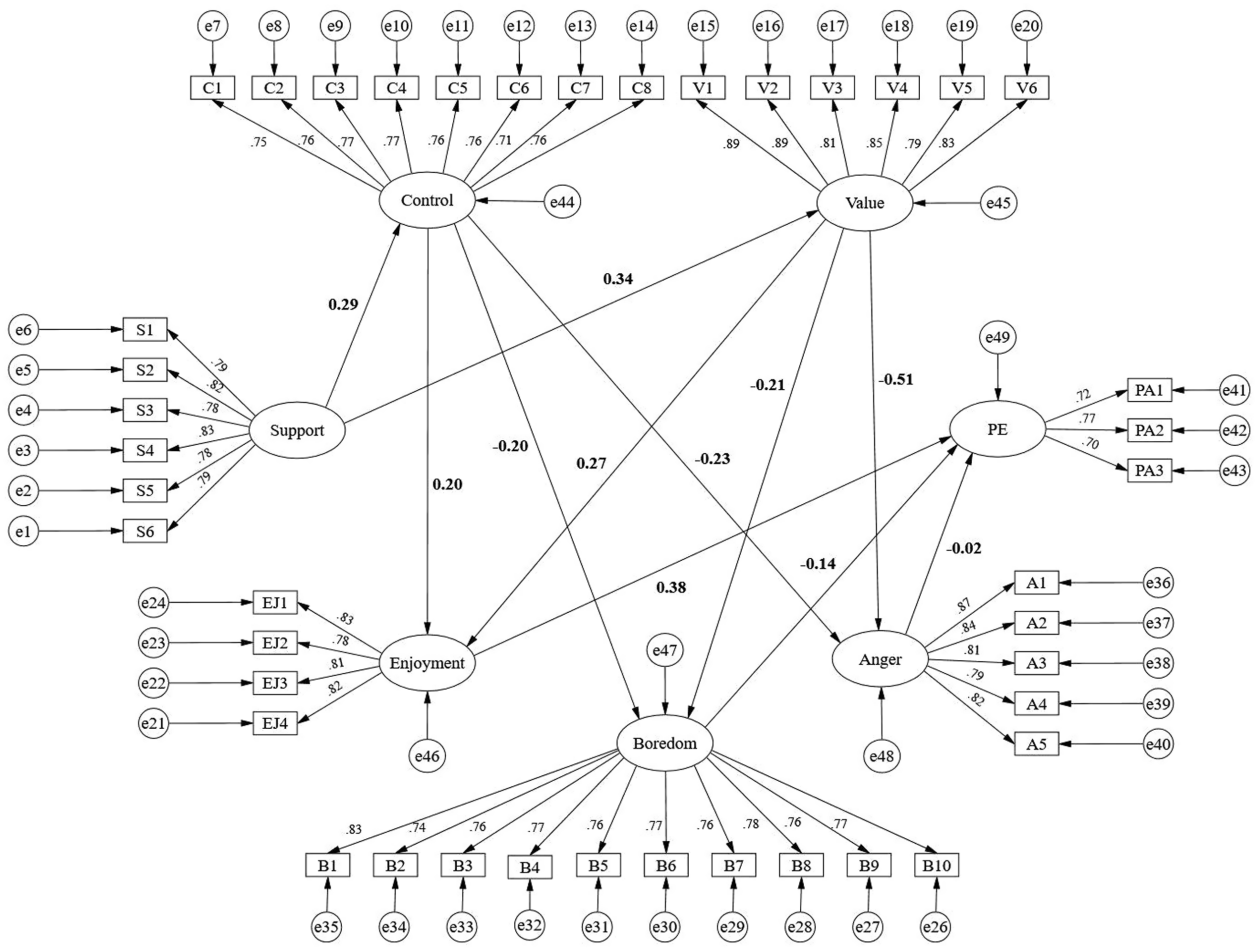
Structural equation model fitting graph.

We applied the Bootstrap method (*N* = 5,000 resamples) to test the model. The results indicated *χ*^2^/df = 3.501, TLI = 0.948, IFI = 0.951, GFI = 0.919, AGFI = 0.909, CFI = 0.951, and RMSEA = 0.041, demonstrating that the structural equation model fit the data very well (see Figure 1). No *post-hoc* model modifications were required, indicating that the hypothesized model was theoretically sound and statistically stable. These results provide strong support for the adequacy of the proposed model structure.

### 4.5 Direct effects

Table 3 shows the direct path way analysis results. Teacher’s autonomy support had a significant positive impact on academic control belief (*β* = 0.290, *p* < 0.001) and subjective task value belief (*β* = 0.339, *p* < 0.001), representing small-to-medium effects. This supports H1 and indicates that autonomy-supportive teaching promotes stronger control and value appraisals among college students in PE.

**Table 3:** Direct path way analysis.

Path	*β*	SE	CR	*p*
Support → control	0.290	0.020	10.122	***
Support → value	0.339	0.030	12.206	***
Control → enjoyment	0.197	0.029	7.042	***
Control → boredom	−0.200	0.028	−7.216	***
Control → anger	−0.235	0.027	−9.490	***
Value → enjoyment	0.267	0.019	9.632	***
Value → boredom	−0.207	0.018	−7.638	***
Value → anger	−0.511	0.018	−20.018	***
Enjoyment → exercise	0.376	0.032	11.615	***
Boredom → exercise	−0.144	0.030	−4.888	***
Anger → exercise	−0.022	0.029	−0.753	0.452

Support = teacher’s autonomy support, control = academic control belief, value = subjective task value belief, exercise = physical exercise behavior, *** indicates *p* < 0.001.

Additionally, college students’ academic control belief and subjective task value belief had a significant positive impact on classroom enjoyment (*β* = 0.197, *β* = 0.267, *p* < 0.001), while they have significantly negative impact on classroom boredom (*β* = −0.200, *β* = −0.207, *p* < 0.001) and classroom anger (*β* = −0.235, *β* = −0.511, *p* < 0.001). The effect of subjective task value belief on anger was medium-to-large, emerging as the strongest cognitive predictor of negative activating emotion. These results support H2 and suggest that students who feel more competent and value PE more highly tend to experience more enjoyment and fewer negative emotions in class.

Thirdly, classroom enjoyment had a significant positive impact on physical activity behavior (*β* = 0.376, *p* < 0.001), with a medium-to-large effect, whereas classroom boredom has a significant negative impact on physical activity behavior (*β* = −0.144, *p* < 0.001). However, classroom anger was the only construct that don not have the predictive effect on physical activity behavior (*β* = −0.022, *p* = 0.452), supporting part of the H3.

### 4.6 Indirect and mediation effects

The Bootstrap method was applied to evaluate the significance of the intermediate effects, with 5,000 resamples. Table 4 summarizes all indirect pathways.

**Table 4:** Indirect effect test.

Path	*β*	95%CI		*p*
		Lower	Upper	
Support—control—enjoyment	0.057	0.036	0.080	***
Support—control—boredom	−0.058	−0.080	−0.040	***
Support—control—anger	−0.068	−0.092	−0.048	***
Support—value—enjoyment	0.090	0.064	0.120	***
Support—value—boredom	−0.070	−0.097	−0.048	***
Support—value—anger	−0.173	−0.215	−0.134	***
Control—enjoyment—exercise	0.074	0.049	0.103	***
Control—boredom—exercise	0.029	0.015	0.046	***
Control—anger—exercise	0.005	−0.009	0.021	0.442
Value—enjoyment—exercise	0.100	0.074	0.131	***
Value—boredom—exercise	0.030	0.016	0.048	***
Value—anger—exercise	0.011	−0.019	0.046	0.451
Support—control—enjoyment—exercise	0.022	0.013	0.032	***
Support—control—boredom—exercise	0.008	0.004	0.014	***
Support—control—anger—exercise	0.002	−0.003	0.006	0.433
Support—value—enjoyment—exercise	0.034	0.023	0.048	***
Support—value—boredom—exercise	0.010	0.005	0.017	***
Support—value—anger—exercise	0.004	−0.006	0.016	0.441

Support = teacher’s autonomy support, control = academic control belief, value = subjective task value belief, exercise = physical exercise behavior, *** indicates *p* < 0.001.

Both academic control belief and subjective task value belief mediated the effect of teacher autonomy support on classroom emotions (H4 supported). Among these pathways, the indirect pathway through subjective task value to enjoyment (0.090, 95% CI [0.064, 0.120]) was the strongest, suggesting that valuing PE plays a particularly important role in shaping students’ positive emotional experiences.

Both enjoyment and boredom further mediated the relationship between control–value appraisals and physical activity behavior (H5 partially supported). The enjoyment-based pathways had medium effects (0.074 and 0.100), larger than the boredom-based pathways (0.029 and 0.030), indicating that increases in positive emotions contribute more strongly to exercise engagement than reductions in negative emotions. Anger-based mediation pathways were non-significant, consistent with the non-significant direct effect of anger.

Finally, the chain mediation effects (teacher autonomy support → control/value appraisals → emotions → physical activity behavior) were also partially supported (H6). Notably, the anger-based chain mediation pathways were non-significant, consistent with the earlier results on anger. The strongest chain pathway was teacher autonomy support → subjective task value → enjoyment → physical activity behavior (0.034, 95% CI [0.023, 0.048]), highlighting the pivotal role of subjective task value in linking autonomy-supportive teaching with positive emotional and behavioral outcomes in PE. Overall, these results demonstrate that autonomy-supportive teaching promotes exercise engagement primarily by strengthening students’ value appraisals and enhancing enjoyment, rather than by reducing negative emotions.

In summary, the hypothesized model was well supported. Autonomy-supportive teaching enhanced students’ control and value appraisals, which in turn shaped their emotional experiences and subsequent exercise behavior. Enjoyment emerged as the most influential emotional predictor of physical activity behavior, outperforming boredom and anger. The mediation findings further indicate that positive emotional pathways contributed more strongly to exercise engagement than negative emotional pathways. These results provide a coherent empirical basis for the theoretical model, laying a clear foundation for the Discussion section.

## 5 Discussion

The present study offers important theoretical contributions by extending the CVTAE to the context of physical education, thereby enriching the understanding of how classroom environments shape students’ emotional and behavioral engagement in physical activity. Consistent with CVTAE, the findings confirmed that teacher autonomy support enhanced students’ control and value appraisals, which subsequently influenced discrete achievement emotions and exercise behavior. This provides empirical support for the applicability of CVTAE beyond traditional academic domains, suggesting that the theory also holds explanatory power in activity-based learning contexts such as physical education, where emotional experiences are often more dynamic and socially situated. Notably, the results revealed that subjective task value exerted a stronger influence on enjoyment than academic control belief, highlighting the centrality of perceived value in shaping positive emotional experiences during PE lessons. This finding adds nuance to existing CVTAE research, which has predominantly emphasized the role of control beliefs in predicting achievement emotions (Li, 2021), and suggests that value appraisals may be particularly salient in contexts where experiential meaning and personal relevance are key drivers of engagement.

Furthermore, the study adds conceptual depth by illustrating how CVTAE can complement motivation-based frameworks such as Self-Determination Theory (SDT). While SDT explains how autonomy-supportive environments nurture intrinsic motivation through the satisfaction of basic psychological needs (Young, 2005), CVTAE clarifies why such environments foster specific emotional experiences that, in turn, promote behavioral adherence. By demonstrating that autonomy-supportive teaching enhances students’ control–value appraisals and elicits positive emotional states that sustain physical activity, this study suggests a conceptual bridge between motivational and emotional processes. Integrating both perspectives provides a more comprehensive account of how teaching practices influence student engagement, indicating that motivation and emotions may operate sequentially to shape exercise behavior. Together, these findings expand the theoretical understanding of emotional mechanisms underlying physical activity participation and offer a more integrated model of how learning environments influence behavioral outcomes.

The findings also align with and extend previous research on achievement emotions and physical activity engagement. Consistent with earlier studies, enjoyment emerged as a key facilitator of exercise participation, reinforcing the notion that positive emotional experiences play a central role in sustaining engagement in physical activity (Xu et al., 2023). Prior research in both Western and Asian contexts has similarly shown that students who experience greater enjoyment during PE are more motivated to continue exercising beyond class time (Leisterer and Jekauc, 2019; Shen et al., 2022). The present results further advance this understanding by revealing that enjoyment exerted a stronger influence on exercise behavior than boredom or anger, highlighting the unique motivational potency of positive affect in physical activity settings.

In contrast, the non-significant predictive effect of anger on exercise behavior diverges from studies conducted in academic subjects, where anger has often been linked to maladaptive engagement patterns (Crisan and Nechita, 2022). One possible explanation is cultural: in collectivist cultures such as China, students tend to suppress or regulate outward expressions of anger to maintain interpersonal harmony, which may reduce its observable behavioral impact (Wei et al., 2013). Additionally, anger may be less salient or less intensely experienced in PE contexts than in academic tasks where frustration, evaluation pressure, or perceived injustice are more likely to trigger such emotions (Chitrakar and Nisanth, 2023). Measurement factors may also play a role, as the anger items in the AEQ were originally developed for classroom academic settings, and subtle forms of irritation or disengagement in PE may not be fully captured. These contextual and cultural distinctions suggest that the emotional mechanisms driving behavior may differ across subject domains and underscore the importance of culturally sensitive and context-specific measurement of negative emotions in future research.

Regarding the mediation pathways, although several indirect effects reached statistical significance, some effect sizes were relatively small, such as the chain mediation of control and boredom. These results should therefore be interpreted with caution in terms of practical impact. Small indirect effects are not uncommon in psychological and educational mediation models, especially when multiple mediators operate sequentially. Nonetheless, the consistent pattern of results suggests that emotional mechanisms—particularly those involving enjoyment—play a meaningful role in translating students’ control–value appraisals into physical activity behavior, even if the magnitude of these effects is modest. Taken together, these findings highlight the importance of examining both the direction and strength of emotional influences and suggest that interventions aimed at fostering positive emotions may produce incremental yet meaningful gains in exercise engagement over time.

The findings of this study offer several practical implications for enhancing physical education instruction and promoting sustained physical activity among college students. First, the results demonstrate the importance of autonomy-supportive teaching practices in cultivating positive emotional experiences and exercise engagement. PE teachers may therefore benefit from adopting instructional approaches that provide students with meaningful choices and a sense of ownership over their learning. For example, teachers can allow students to select from a variety of practice tasks, choose partners or group activities based on preference, or tailor exercise difficulty levels to match their individual readiness. Such practices not only strengthen students’ perceptions of control and value but also foster greater enjoyment during PE lessons. Second, the study highlights the importance of designing learning experiences that enhance the subjective value of physical activities. Teachers can increase task value by clearly communicating the personal, social, and long-term benefits of physical activity, linking PE content to students’ interests and real-life applications, and incorporating goal-setting or self-challenge elements that make learning more meaningful. For instance, integrating sport-based games or project-based modules that connect physical activity to health and lifestyle may help students perceive PE as more relevant and worthwhile. Third, given the substantive role of achievement emotions in influencing exercise behavior, PE instruction should intentionally integrate emotional-support strategies. Beyond promoting enjoyment, teachers should actively minimize boredom by varying class activities, maintaining an optimal level of challenge, and alternating between cooperative and competitive formats to keep students mentally engaged. Providing constructive, empathetic feedback and acknowledging students’ feelings can also help create a psychologically safe learning climate that promotes positive emotional experiences. Such strategies reinforce the emotional pathways identified in this study and can support sustained student engagement in physical activity.

## 6 Limitations and future directions

Despite its contributions, this study is subject to several limitations that should be acknowledged. First, the use of a cross-sectional and self-report design restricts causal inference and may introduce response biases, such as social desirability or cultural suppression of negative emotions, potentially inflating correlations among variables. Future studies should adopt longitudinal, cross-lagged, or experimental designs to verify the temporal ordering and causal mechanisms proposed by the mediation model. Second, although achievement emotions were examined within the framework of CVTAE, only three discrete emotions were included, and the non-significant role of anger may reflect the limited applicability of the AEQ anger subscale in PE contexts or cultural tendencies among Chinese students to regulate anger expression. Future studies should refine emotion measures or incorporate multi-method assessments (e.g., observations or physiological indicators) to capture a broader and more culturally sensitive spectrum of emotions. Third, some of the indirect effects observed in this study were statistically significant but small in magnitude, suggesting that their practical significance should be interpreted with caution. Fourth, while this study controlled for gender, age, and grade, other potentially influential factors—such as personality traits, baseline physical activity, teacher gender, and peer or family support—were not considered and should be included in future models to improve explanatory power. Finally, the findings were based on samples from Chinese universities, which may limit generalizability to other cultural or educational contexts. Cross-cultural research is warranted to examine whether the emotional mechanisms identified here operate similarly in different PE systems and sociocultural environments.

## 7 Conclusion

In conclusion, this study extends the Control–Value Theory of Achievement Emotions (CVTAE) to the physical education context and provides a clearer explanation of how classroom environments shape college students’ exercise behavior. The findings demonstrate that autonomy-supportive teaching enhances students’ control–value appraisals, which in turn evoke specific achievement emotions that influence physical activity engagement. Notably, subjective task value and enjoyment emerged as the key emotional pathways linking teaching practices to sustained exercise participation, highlighting the central role of positive affect in strengthening exercise adherence.

This study contributes to the literature by positioning emotional processes as a bridge between educational environments and exercise motivation. By integrating motivational and emotional mechanisms, the proposed model offers valuable theoretical insight for developing emotionally informed strategies to promote active lifestyles among college students. Future research should examine these mechanisms longitudinally and across diverse cultural and institutional contexts to further validate the model and inform targeted intervention design.

## Data Availability

The raw data supporting the conclusions of this article will be made available by the authors, without undue reservation.

## References

[ref1] Aguirre-Urreta M. I. Hu J. (2019). Detecting common method bias: performance of the Harman's single-factor test. ACM SIGMIS Database Adv. Inf. Syst. 50, 45–70. doi: 10.1145/3330472.3330477

[ref2] Camacho-Morles J. Slemp G. R. Pekrun R. Loderer K. Hou H. Oades L. G. (2021). Activity achievement emotions and academic performance: a meta-analysis. Educ. Psychol. Rev. 33, 1051–1095. doi: 10.1007/s10648-020-09585-3

[ref3] Chan R. C. Lam M. S. (2023). The relationship between perceived school climate, academic engagement, and emotional competence among Chinese students: the moderating role of collectivism. Learn. Individ. Differ. 106:102337. doi: 10.1016/j.lindif.2023.102337

[ref4] Chang H. (2024). The effect of emotional experiences in the physical education classroom on college students' exercise adherence: The mediating role of peer support. Shenyang: Liaoning Normal University.

[ref5] Cheng M. (2024). Study on the influence of new media on college students’ physical exercise behavior. J. Contemp. Educ. Res. 8, 78–82. doi: 10.26689/jcer.v8i3.6421

[ref6] Chitrakar N. Nisanth P. (2023). Frustration and its influences on student motivation and academic performance. Int. J. Sci. Res. Modern Sci. Technol. 2, 01–09. doi: 10.59828/ijsrmst.v2i11.158

[ref7] Crisan S. M. Nechita D. M. (2022). Maladaptive emotion regulation strategies and trait anger as predictors of depression severity. Clin. Psychol. Psychother. 29, 1135–1143. doi: 10.1002/cpp.2702, PMID: 34902882

[ref8] Deliens T. Deforche B. de Bourdeaudhuij I. Clarys P. (2015). Determinants of physical activity and sedentary behaviour in university students: a qualitative study using focus group discussions. BMC Public Health 15:201. doi: 10.1186/s12889-015-1553-4, PMID: 25881120 PMC4349731

[ref9] Duan X. Wang X. Li X. Li S. Zhong Y. Bu T. (2022). Effect of mass sports activity on prosocial behavior: a sequential mediation model of flow trait and subjective wellbeing. Front. Public Health 10:960870. doi: 10.3389/fpubh.2022.960870, PMID: 35979458 PMC9376381

[ref10] Fletcher J. (2016). Applying self-determination theory to college students’ physical-activity behavior: understanding the motivators for physical (in) activity. Commun. Stud. 67, 489–508. doi: 10.1080/10510974.2016.1212911

[ref11] Frenzel A. C. Goetz T. Stockinger K. (2023). “Emotions and emotion regulation” in Handbook of educational psychology (Routledge), 219–244.

[ref12] Gholami Fesharaki M. (2020). Structural equation modeling and its application in psychological studies: a review study. Clin. Psychol. Personality 16, 253–265.

[ref13] Gilchrist P. Wheaton B. (2017). The social benefits of informal and lifestyle sports: a research agenda. Int. J. Sport Policy Polit. 9, 1–10. doi: 10.1080/19406940.2017.1293132

[ref14] Hair JF Jr Hult GT Ringle CM Sarstedt M Danks NP Ray S An introduction to structural equation modeling. Partial least squares structural equation modeling (PLS-SEM) using R: A workbook, 2021: p. 1–29, doi: 10.1007/978-3-030-80519-7_1, PMID: .

[ref16] Huang J. Zhang Y. (2025). A study of the influence of gender stereotypes on the sports participation intention of female college students not majoring in sports in Chongqing colleges and universities. J. Wuhan Instit. Phys. Educ. 59, 94–102.

[ref17] Leisterer S. Jekauc D. (2019). Students’ emotional experience in physical education—a qualitative study for new theoretical insights. Sports 7:10. doi: 10.3390/sports7010010, PMID: 30609809 PMC6359272

[ref18] LI Y. (2021). Traceability of psychological factors in the physical education classroom affecting the amount of extracurricular exercise of students in general colleges and universities - and the mediating effect of achievement emotions. J. Shenyang Sports Inst. 40, 34–42.

[ref19] Li Y.-Y. (2021). Origin of psychological factors in PE class for college students’ extracurricular exercise: also on the mediating effect of achievement emotions. J. Shenyang Sport Univ. 40, 34–42.

[ref20] Li C. (2021). A control–value theory approach to boredom in English classes among university students in China. Mod. Lang. J. 105, 317–334. doi: 10.1111/modl.12693

[ref21] Lv C. Zhang J. Kong I. (2024). A study of influencing factors and intervention strategies of physical activity behaviour of college students based on SDT and TPB. Bull. Sci. Techn. Lit. Sports 32, 201–203+275.

[ref22] Martin J. J. Kulinna P. H. (2004). Self-efficacy theory and the theory of planned behavior: teaching physically active physical education classes. Res. Q. Exerc. Sport 75, 288–297. doi: 10.1080/02701367.2004.10609161, PMID: 15487292

[ref23] Pekrun R. (2006). The control-value theory of achievement emotions: assumptions, corollaries, and implications for educational research and practice. Educ. Psychol. Rev. 18, 315–341. doi: 10.1007/s10648-006-9029-9

[ref24] Pekrun R. (2024). Control-value theory: from achievement emotion to a general theory of human emotions. Educ. Psychol. Rev. 36:83. doi: 10.1007/s10648-024-09909-7

[ref25] Pekrun R. Goetz T. Frenzel A. C. Barchfeld P. Perry R. P. (2011). Measuring emotions in students’ learning and performance: the achievement emotions questionnaire (AEQ). Contemp. Educ. Psychol. 36, 36–48. doi: 10.1016/j.cedpsych.2010.10.002

[ref26] Perry R. P. Hladkyj S. Pekrun R. H. Pelletier S. T. (2001). Academic control and action control in the achievement of college students: a longitudinal field study. J. Educ. Psychol. 93, 776–789. doi: 10.1037/0022-0663.93.4.776

[ref27] Peterson N. E. Sirard J. R. Kulbok P. A. DeBoer M. D. Erickson J. M. (2018). Sedentary behavior and physical activity of young adult university students. Res. Nurs. Health 41, 30–38. doi: 10.1002/nur.21845, PMID: 29315656 PMC10926845

[ref28] Rehman S. Tanwar T. Iram I. Aldabbas M. Veqar Z. (2024). Does regular physical activity protect sleep and mental health of university students: a systematic review. Sleep Vigilance 8, 13–23. doi: 10.1007/s41782-024-00263-w, PMID: 41215982

[ref29] Shen B. Li B. Bo J. (2024). The role of cost in predicting learning outcomes in physical education: an expectancy–value–cost model. J. Teach. Phys. Educ. 1, 1–9.

[ref30] Shen B. Lu X. Bo J. (2022). Cross-cultural studies of motivation in physical education: a systematic review. Int. J. Phys. Act. Health 1:6. doi: 10.18122/ijpah1.1.6.boisestate

[ref31] Simonton K. L. (2021). Testing a model of personal attributes and emotions regarding physical activity and sedentary behaviour. Int. J. Sport Exerc. Psychol. 19, 848–865. doi: 10.1080/1612197X.2020.1739112

[ref32] Simonton K. L. Solmon M. A. Garn A. C. (2021). Exploring perceived autonomy support and emotions in university tennis courses. Int. J. Sport Exerc. Psychol. 19, 134–148. doi: 10.1080/1612197X.2019.1623285

[ref33] Su P. Sheng T. Yue G. (2025). *Analysis of physical activity decision-making and intervention strategies for college students under the perspective of Behavioural Economics.* China school. Health 46, 1070–1073+1078.

[ref34] Teferi G. (2020). The effect of physical activity on academic performance and mental health: systematic review. Am. J. Sci. Eng. Technol. 5:131. doi: 10.11648/j.ajset.20200503.12

[ref35] Wang Q. Xu Z. Tao J. Ma X. (2024). The effects of teacher immediacy and autonomy support on secondary students’ approaches to learning. Educ. Psychol. 44, 78–95. doi: 10.1080/01443410.2024.2302513

[ref36] Wei M. Su J. C. Carrera S. Lin S. P. Yi F. (2013). Suppression and interpersonal harmony: a cross-cultural comparison between Chinese and European Americans. J. Couns. Psychol. 60, 625–633. doi: 10.1037/a0033413, PMID: 23978268

[ref37] Wu X. Liang J. Chen J. Dong W. Lu C. (2024). Physical activity and school adaptation among Chinese junior high school students: chain mediation of resilience and coping styles. Front. Psychol. 15:1376233. doi: 10.3389/fpsyg.2024.1376233, PMID: 38737951 PMC11082357

[ref38] Xiang P. McBride R. Guan J. Solmon M. (2003). Children's motivation in elementary physical education: an expectancy-value model of achievement choice. Res. Q. Exerc. Sport 74, 25–35. doi: 10.1080/02701367.2003.10609061, PMID: 12659473

[ref39] Xu J. Yang J. He D. (2023). Control-value appraisals, academic emotions, and student engagement: a case of Chinese EFL undergraduates. Lang. Teach. Res.:13621688231215276. doi: 10.1177/13621688231215276

[ref40] Yin L. Li F. Si H.-K. (2018). Effect of need support in physical education on adolescent's physical activity in leisure-time:construction and confirmation of a trans-contextual. Sports Sci. 39, 90–100+120.

[ref41] Young M. R. (2005). The motivational effects of the classroom environment in facilitating self-regulated learning. J. Mark. Educ. 27, 25–40. doi: 10.1177/0273475304273346

[ref15] Yuan Y. Tu Y Su Y Jin L Tian Y Chang X . (2025). The mediating effect of self-efficacy and physical activity with the moderating effect of social support on the relationship between negative body image and depression among Chinese college students: a cross-sectional study. BMC Public Health 25:285. doi: 10.1186/s12889-025-21350-1 39849422 PMC11756201

[ref42] Zhang Y. Hasibagen Zhang C. (2022). The influence of social support on the physical exercise behavior of college students: the mediating role of self-efficacy. Front. Psychol. 13:1037518. doi: 10.3389/fpsyg.2022.1037518, PMID: 36532973 PMC9756807

[ref43] Zhang T. Li H. (2023). Structural equation modeling analysis of physical activity behaviour promotion in adolescents - based on the emotional control value theory of achievement. J. Phys. Educ. 30, 67–75.

[ref44] Zimmermann J. Tilga H. Bachner J. Demetriou Y. (2021). The effect of teacher autonomy support on leisure-time physical activity via cognitive appraisals and achievement emotions: a mediation analysis based on the control-value theory. Int. J. Environ. Res. Public Health 18:3987. doi: 10.3390/ijerph18083987, PMID: 33920112 PMC8070009

